# Using Vitek MS v3.0 To Identify Nontuberculous Mycobacteria in Liquid Media in a Clinical Microbiology Laboratory

**DOI:** 10.1128/spectrum.02018-22

**Published:** 2022-10-26

**Authors:** LiuLin Luo, Li Liang, RanRan Zhang, WeiWei Chen, FangYou Yu, Yanlin Zhao, Jun Yue

**Affiliations:** a Department of Clinical Laboratory Medicine, Shanghai Pulmonary Hospital, Tongji University School of Medicine, Shanghai, China; b National Center for Tuberculosis Control and Prevention, Chinese Center for Disease Control and Prevention, Beijing, China; c Department of Tuberculosis, Shanghai Pulmonary Hospital, Tongji University School of Medicine, Shanghai, China; Shenzhen University School of Medicine

**Keywords:** liquid media, MALDI-TOF, mycobacteria, identification

## Abstract

Recently, the incidence of diseases caused by nontuberculous mycobacteria (NTM) has been increasing worldwide, especially in immunocompromised patients and those with potential chronic lung disease. Vitek MS v3.0 matrix-assisted laser desorption ionization–time of flight mass spectrometry (MALDI-TOF MS) has emerged as a rapid and reliable method for identifying mycobacteria in clinical laboratories. This study aimed to evaluate the performance of Vitek MS v3.0 by isolating NTM directly from automated liquid medium systems using patient samples. A total of 855 Mycobacterium growth indicator tube (MGIT)-positive liquid cultures were investigated. Among them, 658 (77.0%) liquid cultures were correctly identified to the species, group, or complex level, 192 (23.0%) resulted in no identification, and 5 (0.6%) were misidentified at the species level. DNA sequencing identified 855 NTM isolates from liquid cultures, comprising 316 isolates of rapidly growing mycobacteria (RGM) and 539 isolates of slow-growing mycobacteria (SGM). Using the Vitek MS system, the RGM integral identification rate (276/316 [87.34%]) was higher than the SGM rate (381/539 [70.69%]) (*P* < 0.01). It was also higher than the SGM rate for all MGIT report-positive periods. These results indicate that the Vitek MS v3.0 system can rapidly identify NTM species from liquid cultures. Further validation using molecular techniques is required.

**IMPORTANCE** Rapid and accurate identification of nontuberculous mycobacteria (NTM) is essential for diagnosis, appropriate therapy, and infection control. Vitek MS v3.0 matrix-assisted laser desorption ionization–time of flight mass spectrometry (MALDI-TOF MS) has emerged as a rapid and reliable method for identifying mycobacteria in clinical laboratories. This study reported a clinical validation of the Vitek MS V3.0 system for identification of NTM isolates from 855 MGIT-positive liquid cultures which contained relatively large NTM types. Vitek MS v3.0 showed a promising rate for identification NTM isolates in positive liquid cultures. Vitek MS v3.0 had a better performance with RGM than with SGM. Vitek MS v3.0 results included “unidentified” or “misidentified” NTM isolates, which would also serve as an important reference for future optimization of this system. Vitek MS v3.0 represented a valuable technique for NTM identification from positive liquid cultures.

## INTRODUCTION

Mycobacterium tuberculosis infections remain a global public health problem, with the World Health Organization (WHO) reporting 9.96 million new cases and 1.21 million related deaths in 2019 (1). In addition to Mycobacterium tuberculosis, a prominent pathogen causing tuberculosis, there are widespread nontuberculous mycobacteria (NTM) that cause human diseases (2, 3). NTM are emerging human pathogens that cause various clinical diseases affecting immunocompromised individuals and those with underlying health conditions. The number of NTM infections has increased dramatically in the past decade due to an increasing number of immunocompromised patients, the implementation of advanced diagnostic tools, and enhanced awareness of the role of NTM in disease (4). Historically, NTM comprise several species, mainly grouped into slow-growing mycobacteria (SGM) and rapidly growing mycobacteria (RGM), according to the growth rate of colonies visible to the naked eye on subculture media in less than or at least 7 days (2). In addition to the growth status, there are differences in infectious disease, treatment regimen, and mycobacterial identification (3). In NTM infections, there is occasionally more than one type of mycobacterial mixed infection. NTM disease and tuberculosis have similar clinical manifestations and are difficult to differentiate, leading to misdiagnosis or loss of the best opportunity for treatment due to untimely diagnosis (5). Rapid diagnosis of NTM disease requires more convenient and faster identification techniques suitable for Mycobacterium species (6).

Rapidly detecting and accurately identifying NTM during the early stages of infection are crucial for the clinical management of patients but can be difficult due to the low growth rates and small differences in the genetic diversity of many species. Conventional identification tools for mycobacterial species, such as microscopy and culture, remain irreplaceable (7). However, the traditional methods of pathogen examination are time-consuming and have poor specificity and sensitivity, and the identification results are not conducive to timely clinical treatment (8). Therefore, clinical mycobacteriology laboratories have developed techniques for accurately and effectively detecting and identifying mycobacteria (9). The Bactec Mycobacterium growth indicator tube (MGIT) 960 system can provide continuous monitoring to identify positive cultures by detecting the fluorescence degree, dramatically reducing the time to diagnose mycobacterial infections (10). The Bactec MGIT 960 is one of the fastest mycobacterial culture, detection, and drug susceptibility measurement systems (11). Additional accurate and rapid identification techniques are required to distinguish MGIT-positive cultures.

Molecular methods, such as PCR-based hybridization and sequencing methods, can provide effective results and good performance for identifying certain species. However, they require technical expertise, specific instruments, and more than 24 h for identification (12, 13). DNA arrays are widely used for rapid identification but are limited to the most commonly isolated mycobacterial species (14). Sequencing methods are reliable and accurate techniques for identifying mycobacteria, even recognizing subspecies. To date, DNA sequencing of the 16S rRNA, *rpoB*, and/or *hsp65* gene remains the gold standard for species identification. However, these methods are costly and time-consuming, require specific equipment and expertise, and are often restricted to reference laboratories (15).

Matrix-assisted laser desorption ionization–time of flight mass spectrometry (MALDI-TOF MS) is adapted for use in microbiology laboratories, where it serves as a paradigm-shifting, rapid, and robust method for accurate microbial identification (16). It has been increasingly used in clinical laboratories to identify bacteria and yeasts (17). Several studies have shown that mycobacterial species can be reliably identified from subculture on a solid-phase medium but may greatly vary from MGIT and other liquid cultures because of the different methodologies and procedures used for protein extraction (18, 19). A recent study by Miller et al. showed improved NTM identification from liquid cultures using the Vitek MS v3.0 system (20), possibly due to the different mycobacterial treatment procedures and MALDI-TOF MS commercial database platforms (14, 20). The purpose of this study was to evaluate the accuracy, robustness, and performance of the Vitek MS v3.0 system to directly identify mycobacterial species isolated from patient samples in liquid media and its usefulness in clinical microbiology laboratories.

## RESULTS

### NTM distribution by sequence-based typing.

During the study period, 11,986 mycobacterial isolates were cultured. Sequence-based typing confirmed that 7.5% (95% confidence interval [CI], 7.04, 7.99) (899/11,986) were NTM. Table 1 summarizes the distribution of NTM species. The most commonly identified organisms were M. avium complex (MAC) (42.5% [95% CI, 39.24, 45.80]; 382/899), followed by the M. abscessus complex (32.2% [95% CI, 29.12, 35.33]; 289/899), M. kansasii (12.4% [95% CI, 10.31, 14.72]; 111/899), M. fortuitum (2.6% [95% CI, 1.67, 3.88]; 23/899), M. gordonae (1.9% [95% CI, 1.14, 3.07]; 17/899), and other NTM species. Mixed isolation from 44 separate patients was confirmed by DNA sequencing (4.9% [95% CI, 3.61, 6.56]; 44/899).

**TABLE 1:** NTM distribution by sequence-based typing in a specialized Chinese tertiary care hospital

Organism(s)	No. (%) of isolates identified by sequencing
M. avium complex	382 (42.5)
M. avium	75 (8.4)
M. intracellulare	271 (30.1)
MAC-X^*a*^	36 (4.0)
*M. chimaera*	2 (0.2)
M. colombiense	5 (0.6)
M. marseillense	26 (2.9)
M. timonense	2 (0.2)
*M. yongonense*	1 (0.1)
M. abscessus/M. chelonae complex	289 (32.2)
M. abscessus	284 (31.6)
M. chelonae	5 (0.6)
M. fortuitum group	23 (2.6)
M. fortuitum	15 (1.7)
*M. peregrinum*	2 (0.2)
M. porcinum	4 (0.5)
*M. septicum*	2 (0.2)
M. kansasii	111 (12.4)
M. gordonae	17 (1.9)
*M. interjectum*	1 (0.1)
*M. asiaticum*	2 (0.2)
*M. lentiflavum*	3 (0.3)
*M. mantenii*	1 (0.1)
*M. parascrofulaceum*	6 (0.7)
*M. seoulense*	1 (0.1)
*M. shigaense*	1 (0.1)
*M. szulgai*	3 (0.3)
*M. triplex*	2 (0.2)
M. xenopi	7 (0.8)
*M. angelicum*	2 (0.2)
*M. monacense*	2 (0.2)
*M. neoaurum*	1 (0.1)
*M. wolinskyi*	1 (0.1)
M. abscessus + M. kansasii	3 (0.3)
M. abscessus + M. avium	4 (0.5)
M. abscessus + M. intracellulare	17 (1.9)
M. intracellulare + M. kansasii	4 (0.5)
M. intracellulare + M. tuberculosis complex	6 (0.7)
M. avium + M. tuberculosis complex	3 (0.3)
M. abscessus + M. tuberculosis complex	2 (0.2)
M. kansasii + M. tuberculosis complex	1 (0.1)
M. abscessus *+* Nocardia farcinica	2 (0.2)
M. intracellulare *+* Nocardia farcinica	1 (0.1)
M. mucogenicum *+* Nocardia farcinica	1 (0.1)
Total	899 (100)

a MAC-X includes *M. chimaera*, M. colombiense, M. arosiense, M. vulneris, M. bouchedurhonense, M. marseillense, M. timonense, M. paraintracellulare, and *M. yongonense*.

After sequence-based typing, we categorized 18 species from the 33 isolates that were relatively uncommon in China into species. We found M. interjectum (0.1%; 1/899), M. asiaticum (0.2%; 2/899), M. mantenii (0.1%; 1/899), M. parascrofulaceum (0.7%; 6/899), M. seoulense (0.1%; 1/899), M. shigaense (0.1%; 1/899), M. angelicum (0.2%; 2/899), M. monacense (0.2%; 2/899), M. neoaurum (0.1%; 1/899), and M. wolinskyi (0.1%; 1/899). We found coinfection with M. tuberculosis or other NTM species, such as M. abscessus, M. kansasii, M. avium, M. intracellulare, and M. mucogenicum, in a minority of cases (4.9% [95% CI, 3.61, 6.56]; 44/899).

### Overall performance for NTM identification.

MALDI-TOF MS was compared with gene sequencing of 899 MGIT-positive liquid cultures from patient specimens. We extracted and tested 899 MGIT-positive liquid cultures using the Vitek MS system. All MGIT-positive cultures were grown on Middlebrook 7H10 medium, and among 899 liquid culture specimens, DNA sequencing identified 855 as monomicrobial and 44 as polymicrobial. DNA sequencing recognized 855 NTM isolates from liquid cultures, comprising 29 species. Only 15 species were included in the Vitek MS v3.0 database (the species identified by the Vitek MS v3.0 database in the supplemental material). Among these isolates, 316 were RGM and 539 were SGM. A total of 658/855 (77.0% [95% CI, 73.96, 79.71]) liquid cultures were correctly identified to the species, group, or complex level, and 197/855 (23.0% [95% CI, 20.29, 26.04]) were unidentified by the Vitek MS system. Table 2 presents the results.

**TABLE 2:** Vitek MS v3.0 library performance for identifying Mycobacterium spp. in liquid medium

Reference ID^*a*^	No. (%) of isolates with indicated Vitek MS v3.0 ID results according to identification category (confidence value)			
	Excellent (≥99.9%)	Good (60.0–<99.9%)	No ID (<60.0%)	Mis-ID
NTM slow growers				
M. avium complex				
M. avium (*n* = 75)	51 (68)	9 (12)	15 (20)	
M. intracellulare (*n* = 271)	203 (67.2)	21 (7.7)	68 (25.1)	
MAC-X^*c*^ (*n* = 36) (includes but may not discriminate among M. colombiense*, M. marseillense*, M. timonense*, M. chimaera*, and M. yongonense*)	11 (30.6)		25 (69.4)	
M. angelicum* (*n* = 2)			2 (100)	
*M. asiaticum* (*n* = 2)			2 (100)	
M. gordonae (*n* = 17)	11 (64.7)	1 (5.9)	5 (29.4)	
M. interjectum* (*n* = 1)			1 (100)	
M. kansasii (*n* = 111)	80 (72.1)	8 (7.2)	23 (20.7)	
*M. lentiflavum* (*n* = 3)	3 (100)			
*M. mantenii* (*n* = 1)			1 (100)	
M. parascrofulaceum* (*n* = 6)			1 (16.7)	5 (83.3)
M. seoulense* (*n* = 1)			1 (100)	
M. shigaense* (*n* = 1)			1 (100)	
*M. szulgai* (*n* = 3)	3 (100)			
*M. triplex* (*n* = 2)			2 (100)	
M. xenopi (*n* = 7)	2 (28.6)		5 (71.4)	
NTM rapid growers				
M. abscessus (*n* = 284)	244 (85.9)	9 (3.6)	31 (10.9)	
M. chelonae (*n* = 5)	4 (80)		1 (20)	
M. fortuitum group (*n* = 23) (includes but does not discriminate between M. porcinum, *M. peregrinum*, and M. septicum*)	18 (78.3)^*b*^		5 (21.7)	
M. monacense* (*n* = 2)			2 (100)	
*M. neoaurum* (*n* = 1)	1 (100)			
M. wolinskyi* (*n* = 1)			1 (100)	
Total (*n* = 855)	610 (71.4)	48 (5.6)	192 (22.4)	5 (0.6)

a Species not included in the bioMérieux Vitek MS v3.0 database are indicated by asterisks. ID, identification.

b Identification results correct to complex or group level.

c MAC-X includes *M. chimaera*, M. colombiense, M. arosiense, M. vulneris, M. bouchedurhonense, M. marseillense, M. timonense, M. paraintracellulare, and *M. yongonense*.

RGM included nine species with a total identification rate of 276/316 (87.34% [95% CI, 83.04, 90.70]) in liquid cultures, and M. abscessus was the most frequently encountered species, with an identification rate of 253/284 (89.1% [95% CI, 84.72, 92.35]). For the M. fortuitum group, 18/23 (78.3% [95% CI, 55.79, 91.71]) cultures were correctly identified at the group level. The less frequently encountered RGM species were M. chelonae (4/5, or 80% [95% CI, 29.88, 98.95]), *M. monacense* (0/2, or 0%), *M. neoaurum* (1/1, or 100%), and *M. wolinskyi* (0/1, or 0%).

SGM included 20 species, with a total identification rate of 384/539 (72.2% [95% CI, 67.18, 74.99]) in liquid cultures. Vitek MS v3.0 successfully identified the most important MAC cultures, including M. intracellulare (203/271, or 74.9% [95% CI, 69.23, 79.87]) and M. avium (60/75, or 80.0% [95% CI, 68.85, 88.02]). Other MAC complex cultures were identified to the complex level rather than the species level because the Vitek MS v3.0 database did not include them. These complexes included M. colombiense, M. marseillense, M. timonense, M. chimaera, and M. yongonense, and a total of 11/36 (30.6% [95% CI, 16.92, 48.27]) isolates were correctly identified at the complex level. In addition, M. parascrofulaceum was misidentified as M. scrofulaceum. Other identification results included M. gordonae (12/17, or 70.6% [95% CI, 44.05, 88.62]), *M. angelicum* (0/2, or 0%), *M. interjectum* (0/1, or 0%), *M. mantenii* (0/1, or 0%), *M. seoulens* (0/1, or 0%), *M. shigaense* (0/1, or 0%), M. lentiflavum (3/3, or 100%), M. xenopi (2/7, or 28.6% [95% CI, 5.11, 69.74]), *M. asiaticum* (0/2, or 0%), *M. neoaurum* (1/1, or 100%), M. szulgai (3/3, or 100%), and M. triplex (0/2, or 0%).

During the Vitek MS v3.0 evaluation in liquid medium, 576/855 (67.4% [95% CI, 64.10, 70.49]) isolates were initially identified, and 82/855 (9.6% [95% CI, 7.74, 11.61]) additional isolates were identified on repeat testing, with 192/855 (22.4% [95% CI, 19.73, 25.44]) isolates remaining unidentified and 5/855 (0.6% [95% CI, 0.21, 1.44]) isolates misidentified. All results are shown in Table 3.

**TABLE 3:** Vitek MS v3.0 library performance for identifying Mycobacterium spp. in liquid medium on the first and second runs

Organism(s)^*a*^	No. (%) of isolates identified					
	Total	v3.0	1^*b*^	2^*c*^	No ID	Mis-ID
NTM slow growers						
M. avium complex						
M. avium	75	60 (80)	56	4	15	0
M. intracellulare	271	224 (74.9)	214	10	68	0
MAC-X^*d*^ (includes but may not discriminate among M. colombiense*, M. marseillense*, M. timonense*, M. chimaera*, and M. yongonense*)	36	11 (30.6)	11	0	25	0
M. angelicum*	2	0 (0.0)	0	0	2	0
*M. asiaticum*	2	0 (0.0)	0	0	2	0
M. gordonae	17	12 (70.6)	11	1	5	0
M. interjectum*	1	0 (0.0)	0	0	1	0
M. kansasii	111	88 (79.3)	82	6	23	0
*M. lentiflavum*	3	3 (100)	0	0	0	0
*M. mantenii*	1	0 (0.0)	0	0	1	
M. parascrofulaceum*	6	0 (0.0)	0	0	1	5
M. seoulense*	1	0 (0.0)	0	0	1	0
M. shigaense*	1	0 (0.0)	0	0	1	0
*M. szulgai*	3	3 (100)	3	0	0	0
*M. triplex*	2	0 (0.0)	0	0	2	0
M. xenopi	7	2 (28.6)	2	0	5	0
NTM rapid growers						
M. abscessus	284	253 (89.1)	193	60	31	0
M. chelonae	5	4 (80)	4	0	1	0
M. fortuitum group (includes but does not discriminate between M. porcinum, *M. peregrinum*, M. septicum*)	23	18 (78.3)^*e*^	17	1	5	0
M. monacense*	2	0 (0.0)	0	0	2	0
*M. neoaurum*	1	1 (100)	1	0	0	0
M. wolinskyi*	1	0 (0.0)	0	0	1	0
Total	855	658 (77.0)	576 (67.4)	82 (9.6)	192 (22.4)	5 (0.6)

a Species not included in the bioMérieux Vitek MS v3.0 database are indicated by asterisks.

b 1, number of strains identified for the first time.

c 2, number of strains identified for the second time.

d MAC-X includes *M. chimaera*, M. colombiense, M. arosiense, M. vulneris, M. bouchedurhonense, M. marseillense, M. timonense, M. paraintracellulare, and *M. yongonense*.

e Identification results correct to complex or group level.

### Performance of Vitek MS in identifying RGM and SGM at different MGIT report-positive days.

A total of 29 mycobacterial species (855 isolates), comprising 9 RGM species (316 isolates) and 20 SGM species (539 isolates), were used for analysis. Among the RGM species, six species were found in the bioMérieux Vitek MS v3.0 database, and three species were absent. Among the SGM species, 9 species existed in the Vitek MS v3.0 database and 11 species did not. We analyzed the identification rate of these Mycobacterium isolates according to the different periods of MGIT report-positive days (Table 4).

**TABLE 4:** Vitek MS performance for identifying RGM and SGM at different MGIT report-positive days

Organism(s)^*a*^	Avg report-positive days	No. (%) of isolates identified at different MGIT report-positive days					
		Total	0 < d ≤ 6	6 < d ≤ 11	11 < d ≤ 16	16 < d ≤ 21	d > 21
RGM							
M. abscessus	5.57 ± 3.85	253/284 (89.08%)	202/225 (89.78)	29/35 (82.86)	13/14 (92.86)	7/7 (100)	2/3 (66.67)
M. chelonae	11.62 ± 4.33	4/5 (80)	0	3/3 (100)	1/1 (100)	0/1 (0)	0
M. fortuitum group	7.72 ± 5.75	18/23 (78.26)	10/13 (76.92)	4/6 (66.67)	2/2 (100)	0	2/2 (100)
M. fortuitum	6.67 ± 4.62	11/15 (73.33)	8/10 (80)	1/3 (33.33)	1/1 (100)	0	1/1 (100)
*M. peregrinum*	7.77 ± 1.15	2/2 (100)	0	2/2 (100)	0	0	0
M. porcinum	9.99 ± 9.04	4/4 (100)	2/2 (100)	1/1 (100)	0	0	1/1 (100)
M. septicum*	10.27 ± 3.90	1/2 (50)	0/1 (0)	0	1/1 (100)	0	0
M. monacense*	4.38 ± 5.42	0/2 (0)	0/1 (100)	0/1 (100)	0	0	0
*M. neoaurum*	6.04	1/1 (100)	0	1/1 (100)	0	0	0
M. wolinskyi*	5.67	0/1 (0)	0/1 (0)	0	0	0	0
Identification rate	5.82 ± 4.11	276/316 (87.34)	212/240 (88.33)	37/46 (80.43)	16/17 (94.12)	7/8 (87.5)	4/5 (80)
Identification rate^*b*^	6.04 ± 3.90	275/311 (88.42)	212/237 (89.45）	37/45 (82.22)	15/16 (93.75)	7/8 (87.5)	4/5 (80)
SGM							
M. avium complex							
M. avium	11.63 ± 7.05	60/75 (80.0)	11/18 (61.12)	18/20 (90)	23/27 (85.19)	5/6 (83.33)	3/4 (75)
M. intracellulare	8.73 ± 5.85	203/271 (74.91)	82/124 (66.13)	63/77 (81.82)	39/46 (84.78)	8/13 (61.54)	11/11 (100)
MAC-X*	12.81 ± 7.40	11/36 (30.55)	2/6 (33.33)	4/12 (33.33)	3/11 (27.27)	1/2 (50)	1/5 (20)
M. chimaera*	22.33 ± 17.32	0/2 (0)	0	0/1 (0)	0	0	0/1 (0)
M. colombiense*	11.72 ± 2.85	1/5 (20)	0	1/2 (50)	0/3 (0)	0	0
M. marseillense*	13.16 ± 8.31	10/26 (38.46)	2/6 (33.3)	3/6 (20)	3/8 (37.5)	1/2 (50)	1/4 (25)
M. timonense*	9.33 ± 0.24	0/2 (0)	0	0/2 (0)	0	0	0
M. yongonense*	7.96	0/1 (0)	0	0/1 (0)	0	0	0
M. angelicum*	4.4 ± 0.21	0/2 (0)	0/2 (0)	0	0	0	0
*M. asiaticum*	21.77 ± 6.69	0/2 (0)	0	0	0	0/1 (0)	0/1 (0)
M. gordonae	18.74 ± 8.63	12/17 (70.59)	0/1 (0)	1/1 (100)	5/8 (62.5)	1/2 (50)	5/5 (100)
M. interjectum*	15.08	0/1 (0)	0	0	0/1 (0)	0	0
M. kansasii	12.19 ± 6.67	88/111 (79.28)	16/20 (80)	28/38 (73.68)	19/23 (82.61)	14/17 (82.35)	11/13 (84.62)
*M. lentiflavum*	30.35 ± 5.69	2/3 (66.7）	0	0	0	0	2/3 (66.67)
M. mantenii*	23.21	0/1 (0)	0	0	0	0	0/1 (0)
M. parascrofulaceum*	25.55 ± 9.53	0/6 (0)	0	0/1 (100)	0	0	0/5 (0)
M. seoulense*	20.75	0/1 (0)	0	0	0	0/1 (0)	0
M. shigaense*	31.04	0/1 (0)	0	0	0	0	0/1 (0)
*M. szulgai*	13.32 ± 10.60	3/3 (100)	0	2/2 (100)	0	0	1/1 (100)
*M. triplex*	8.13 ± 0.29	0/2 (0)	0	0/2 (0)	0	0	0
M. xenopi	24.28 ± 17.29	2/7 (28.57)	1/2 (50)	1/1 (100)	0	0	0/4 (0)
Identification rate	11.12 ± 7.66	381/539 (70.69)	112/173 (64.74)	117/154 (75.97)	89/116 (76.72)	29/42 (69.05)	34/54 (62.96)
Identification rate^*b*^	10.85 ± 6.52	370/491 (75.36)	110/165 (66.67)	113/141 (80.14)	86/104 (82.69)	28/39 (71.79)	33/42 (78.57)

a Species not included in the bioMérieux Vitek MS v3.0 database are indicated by asterisks.

b Identification rate after excluding species not in the bioMérieux Vitek MS database.

For the RGM, the average MGIT report-positive days were 5.82 ± 4.11. The integral rate of identification to the species, group, or complex level was 276/316 (87.34% [95% CI, 83.04, 90.70]), and the identification rate after excluding species not in the bioMérieux Vitek MS v3.0 database was 275/311 (88.42% [95% CI, 84.21, 91.65]).

M. abscessus and M. fortuitum groups were the most frequently encountered species of RGM, with average MGIT report-positive days of 5.57 ± 3.85 and 7.72 ± 5.75 and identification rates of 253/284 (89.08% [95% CI, 84.72, 92.35]) and 18/23 (78.26% [95% CI, 55.79, 91.71]), respectively. Other subspecies of M. abscessus were not tested. The M. fortuitum group was identified to the group level (includes but does not discriminate between M. fortuitum, M. porcinum, M. peregrinum, and M. septicum). Of the other RGM species with a few isolates, all of the M. chelonae strains (with average MGIT report-positive days at 11.62 ± 4.33; 4/5, or 80% [95% CI, 29.88, 98.25]) were correctly identified to the species level. *M. monacense* (two isolates) and *M. wolinskyi* (one isolate) were not included in the Vitek MS database, and all were unidentified. One *M. neoaurum* isolate was correctly identified between days 6 and 11 (6 < d ≤ 11 days).

For SGM, the average number of MGIT report-positive days was 11.12 ± 7.66. The integral rate of identification to the species, group, or complex level was 381/539 (70.69% [95% CI, 66.62, 74.46]), and the identification rate after excluding species not included in the Vitek MS v3.0 database was 370/491 (75.36% [95% CI, 71.26, 79.06]).

M. avium and M. intracellulare were the two most encountered members of the MAC, with average MGIT report-positive days at 11.63 ± 7.05 and 8.73 ± 5.85 and identification rates at 60/75 (80.0% [95% CI, 68.85, 88.02]) and 203/271 (74.91% [95% CI, 69.23, 79.87]), respectively. “MAC-X” refers to new species identification and classification within MAC at the molecular level, such as *M. chimaera*, M. colombiense, M. arosiense, M. vulneris, M. bouchedurhonense, M. marseillense, M. timonense, M. paraintracellulare, and *M. yongonense*. MAC-X, as with other MAC members in this study, was identified to the complex rather than the species level, generally as a result of being absent from the Vitek MS v3.0 database. The integral MAC-X identification rate was 11/36 (30.55% [95% CI, 16.92, 48.27]), with average MGIT report-positive days of 12.81 ± 7.40. For M. colombiense, one of five (20%) isolates were correctly identified to the complex level as M. avium at 6 <d ≤ 11 days. For M. marseillense, 10/26 (38.46% [95% CI, 20.91, 59.27]) were correctly identified to the complex level as M. intracellulare. The integral identification rate for M. kansasii was 88/111 (79.28% [95% CI, 70.33, 86.16]), with average MGIT report-positive days of 12.19 ± 6.67. The integral identification rate for M. gordonae was 12/17 (70.59% [95% CI, 44.05, 88.62]), with average MGIT report-positive days at 18.74 ± 8.63. The other SGM members, *M. asiaticum* (both of 2 isolates were unidentified), *M. lentiflavum* (2 of 3 isolates were identified at d > 21 days), and *M. szulgai* (2/2 [100%]), were correctly identified at 16 < d ≤ 21 days and 1/1 (100%) was correctly identified at d > 21 days. For *M. triplex* (both of 2 isolates were unidentified) and M. xenopi (1 of 2 isolates was identified at 0 < d ≤ 6 days), 1/1 (100%) was identified at 6 < d ≤ 11 days and 4 isolates were unidentified at d > 21 days. Some species not included in the Vitek MS database, comprising *M. angelicum* (two isolates), *M. interjectum* (one isolate), *M. mantenii* (one isolate), *M. seoulense* (one isolate), and *M. shigaense* (one isolate), were unidentified, and five of six *M*. *parascrofulaceum* isolates were misidentified as M. scrofulaceum (Table 4).

We analyzed the identification rate between the RGM and SGM and found a significant difference in the integral identification rate (*P* < 0.01; χ^2^ = 28.758). After excluding the species not found in the Vitek MS v3.0 database, there was a significant difference in the integral identification rate (*P* < 0.01; χ^2^ = 20.653) between RGM and SGM. We compared the MALDI-TOF MS RGM and SGM identification rates and found that the integral identification rate of RGM (276/316, or 87.34% [95% CI, 83.04, 90.70]) was higher than that of SGM (381/539, or 70.69% [95% CI, 66.62, 74.46]) (*P* < 0.01). We excluded the species not included in the database to objectively evaluate the identification effect of Vitek MS v3.0. The identification rates were elevated, and the integral identification rate of RGM (275/311, or 88.42% [95% CI, 84.21, 91.65]) was higher than that of SGM (370/491, or 75.36% [95% CI, 71.26, 79.06]) (*P* < 0.01). Comparison of the identification rate between RGM and SGM at different MGIT report-positive days revealed that the identification rate was higher in RGM than SGM, at 0 < d ≤ 6 MGIT report-positive days, and the difference was statistically significant (*P* < 0.01). In the other MGIT report-positive period, the RGM identification rate was higher than for SGM, but the difference was not statistically significant. After exclusion of the species not in the database, the integral identification rate in RGM was still higher than that in SGM, with a significant statistical difference (*P* < 0.01). The integral identification rate in RGM was higher than in SGM at all the MGIT report-positive periods, but a statistically significant difference only existed at 0 < d ≤ 6 days (*P* < 0.01).

### Performance for identifying polymicrobial infection in a liquid medium.

All MGIT-positive cultures were grown on Middlebrook 7H10 medium, and all isolates from liquid culture specimens were identified by DNA sequencing. Of the 899 liquid cultures, 855 were monomicrobial (95.1% [95% CI, 93.44, 96.39]) and 44 were polymicrobial (4.9% [95% CI, 3.61, 6.56]). Among all cases, 24/44 were M. abscessus mixed with other NTM species, especially M. intracellulare. In addition, 12/44 were NTM mixed with M. tuberculosis, and 4/44 were NTM mixed with Nocardia farcinica. Among these polymicrobial cultures, the Vitek MS v3.0 system identified one of the species for the majority (33/44, or 75% [95% CI, 59.35, 86.30]) of the liquid culture specimens tested. The average MGIT report-positive days was 10.2, compared to 13.85 in monomicrobial cultures. Table 5 provides additional details.

**TABLE 5:** Vitek MS performance for identifying polymicrobial cultures

Reference identification	MS identification of MGIT broth	MGIT report-positive days	Total
M. abscessus + NTM			
M. abscessus	1/4	9.82	4
M. avium	3/4		
M. abscessus	0	6.53	3
M. kansasii	2/3		
M. abscessus	5/17	6.53	17
M. intracellulare	8/17		
M. intracellulare	1/4	9.36	4
M. kansasii	2/4		
NTM + *Nocardia*			
NTM M. intracellulare M. mucogenicum M. abscessus	0/4	10.69	1 1 2
Nocardia farcinica	4/4		
NTM + M. tuberculosis complex			
NTM M. intracellulare M. avium M. abscessus M. kansasii	3/12	14.37	6 3 2 1
M. tuberculosis complex	5/12		
Total		10.24	44

### Unidentified and misidentified isolates in liquid medium by Vitek MS.

The Vitek MS v3.0 system left unidentified and misidentified 195 isolates from MGIT-positive cultures representing 23 different NTM species. Of these, 39 isolates representing 13 NTM species were not in the Vitek MS v3.0 database, and another 156 isolates representing 10 NTM species were included in the database. Among these unidentified isolates, 189/195 were left unidentified, while 6 *M. parascrofulaceum* isolates were misidentified as M. scrofulaceum. Table 6 presents these results.

**TABLE 6:** Analysis of the discrepant strains

Reference identification	v3.0 identification	v3.0 database	Target sequenced
M. abscessus (*n* = 31)	No ID	Yes	16S RNA and *hsp65*
M. intracellulare (*n* = 68)	No ID	Yes	16S RNA
M. kansasii (*n* = 23)	No ID	Yes	16S RNA and *hsp65*
M. avium (*n* = 15)	No ID	Yes	16S RNA
M. fortuitum (*n* = 4)	No ID	Yes	16S RNA and *hsp65*
*M. septicum* (*n* = 1)	No ID	No	16S RNA and *hsp65*
M. gordonae (*n* = 5)	No ID	Yes	16S RNA
M. chelonae (*n* = 1)	No ID	Yes	16S RNA
*M. parascrofulaceum* (*n* = 6)	M. scrofulaceum	No	16S RNA
M. xenopi (*n* = 5)	No ID	Yes	16S RNA
M. marseillense (*n* = 16)	No ID	No	16S RNA
M. colombiense (*n* = 4)	No ID	No	16S RNA
*M. angelicum* (*n* = 2)	No ID	No	16S RNA
*M. asiaticum* (*n* = 2)	No ID	Yes	16S RNA
*M. chimaera* (*n* = 2)	No ID	No	16S RNA
*M. interjectum* (*n* = 1)	No ID	No	16S RNA
*M. mantenii* (*n* = 1)	No ID	No	16S RNA
*M. monacense* (*n* = 2)	No ID	No	16S RNA
*M. seoulense* (*n* = 1)	No ID	No	16S RNA
*M. shigaense* (*n* = 1)	No ID	No	16S RNA
*M. triplex* (*n* = 2)	No ID	Yes	16S RNA
*M. wolinskyi* (*n* = 1)	No ID	No	16S RNA
*M. yongonense* (*n* = 1)	No ID	No	16S RNA

## DISCUSSION

Implementing MALDI-TOF MS to identify microorganisms has revolutionized workflow and improved turnaround time in clinical microbiology laboratories. This technology generates characteristic mass spectral fingerprints which are compared with a large library of mass spectra. As the spectral fingerprints are unique signatures for each microorganism, accurate microbial identification at the genus and species levels is done using bioinformatics pattern profiling.

Recently, several studies have evaluated the effectiveness of this method for recognizing NTM isolates (15, 20). Direct identification of mycobacteria from a positive liquid culture through an automated detection system can substantially reduce the turnaround time to diagnosis compared to those of conventional microbiological methods. It has been reported that the identification rate of MALDI-TOF MS in a culture broth is much lower than that of 7H11 Middlebrook or LJ medium, as the pretreatment is more tedious (15, 20). This study included 855 NTM isolates from liquid cultures to assess the performance of Vitek MS v3.0 and the relationship between the identification rate of different NTM species. The overall level of correct NTM identification in the positive liquid cultures was 77.2%, with only 0.6% (5/855) misidentified samples. Miller et al. obtained similar results with clinical liquid cultures, reporting 87.7% correct identification when testing in MGITs (20).

Vitek MS v3.0 had no misidentified samples at the genus level and could differentiate some closely related species. However, it failed to distinguish between MAC and M. fortuitum group members. For SGM, the MAC and M. kansasii are the most frequently reported clinically significant species (21). M. colombiense, M. marseillense, and M. timonense belong to the MAC and were identified as M. intracellulare because of their close phylogenetic relationships (22). Although the Vitek MS V3.0 correctly identified all M. intracellulare isolates, other members of the M. avium complex could not be reliably differentiated. This can be problematic for species not included in the database that are closely related to those included in the database, such as M. colombiense, as they may be misidentified as a similar species rather than not identified. It is worth mentioning that the misidentifications detected always involved closely related species and they would have led to a different therapy for the patient. Reporting identifications to the complex or group level may be required other molecular methods for a final identification. M. porcinum, *M. peregrinum*, and M. septicum are included and were identified as part of the M. fortuitum group. These isolates were misidentified at the species level but were correctly identified at the complex level, generally as a result of not being included in the Vitek database. *M. parascrofulaceum* is an opportunistic pathogen (23). However, the majority of the isolates (5/6) were misidentified as M. scrofulaceum in our study. In the future, adding *M. parascrofulaceum* and other MAC and M. fortuitum species to the database will be considered.

In this study, there was a significant difference in the average MGIT report-positive days between RGM and SGM isolates. The average MGIT report days were 5.82 ± 4.11 and 11.12 ± 7.66 for RGM and SGM, respectively. The mean MGIT report-positive days in RGM were shorter than those in SGM, probably due to the different growth rates and amounts of these mycobacteria (24). In this study, the identification of RGM by Vitek MS v3.0 had a better effect than with SGM, especially at 0 < d ≤ 6 MGIT report-positive days. A similar study demonstrated that the ability of MALDI-TOF MS to identify SGM is not as good as the ability to identify RGM (25, 26).

We analyzed the distribution and identification rate among different MGIT report-positive days in RGM and SGM. For RGM, the majority of isolates (240/316) were distributed in the MGIT report-positive period at 0 < d ≤ 6 days and a minority were distributed in more than 6 days. The identification rates in RGM were successful, without a significant statistical difference among different MGIT report-positive periods. A maximum of 6 days of MGIT report-positive time was enough to ensure that RGM did not enter the stationary phase since the Vitek MS v3.0 database was developed on protein profiles from the log phase. In SGM, the majority of isolates were distributed in the MGIT report-positive period at 0 < d ≤ 6 days (173/539), 6 < d ≤ 11 days (154/539), and 11 < d ≤ 16 days (116/539). The identification rate was higher at 6 < d ≤ 11 and 11 < d ≤ 16 MGIT report-positive days than for the other periods, with a significant statistical difference (*P* = 0.018). This implied that SGM identification at 6 < d ≤ 11 and 11 < d ≤ 16 MGIT report-positive days by Vitek MS v3.0 had a better effect than other MGIT report-positive periods. The identified difference at different MGIT report-positive days was probably associated with the growth status and amount of bacteria. Although the reasons for the low identification rate with SGM were not clear, we propose that the low bacterial load of the log phase combined with interfering substances present in MGIT tubes reduced the production of sufficient protein peak levels for identification. It is still problematic for species not included in the Vitek MS v3.0 database (15). Due to the limited ability of Vitek MS v3.0 to identify SGM from MGIT-positive cultures, additional accurate and rapid molecular techniques are required.

There were 195 isolates that were left unidentified or misidentified, while the majority of them (156/195) were actually included in the database of Vitek MS v3.0. Although the reasons for the failure of detection of these isolates with Vitek MS v3.0 are not clear, on the one hand, we suspect that the low organism burden combined with the interfering substances present in MGITs reduces ability to generate sufficient protein peaks for identification. To obtain enough biomass for MALDI-TOF MS analysis, the technologist may have optimized sample processing method. On the other hand, insufficiency of the database is considered. To improve the utility of MALDI-TOF MS for NTM identification, further attention should be paid to development of databases that include spectra obtained from different growth conditions.

MALDI-TOF MS was intended to identify pure cultures. Mixed Mycobacterium cultures present a problem for MALDI-TOF MS. In this study, the proportion of polymicrobial cultures among the total number of cultures was 4.9% (44/899). Among these polymicrobial cultures, the Vitek MS v3.0 system only provided a single correct identification of the species for the majority (33/44, or 75%) of the liquid culture specimens tested. We suggest that mixed NTM cultures are not uncommon and that all mycobacterial species should be identified because the species may have clinical significance in treatment. The majority of NTM isolated in NTM mixed cultures are species that are pathogenic and known to cause lung diseases (21). Unfortunately, MALDI-TOF MS often identified one species from mixed cultures of two species, and the identification result also depended on the concentration and protein content of the RGM. Molecular methods allow the simultaneous identification of two NTM species in respiratory specimens when a mixed culture is suspected.

Vitek MS v3.0 is more cost-effective and provides faster identification of mycobacterial isolates to the species level than currently used methods. Shortening the time period required to identify an NTM from days to minutes should improve clinical outcomes. The lower cost in comparison to current identification methods is another advantage of this technology, despite the initial investment in expensive equipment (22, 24, 27). The gold standard for the identification of mycobacteria is DNA sequencing. MALDI-TOF MS is faster (by approximately 1 day), is more cost-effective, and is less labor-intensive than sequencing. Since implementing MALDI-TOF MS for these organisms, the number of isolates that have required sequencing for a final identification has decreased by approximately 80% in our laboratory. As sequencing technology becomes faster and less expensive, it will certainly play an important role in the identification of NTM.

The strengths of this study were the large number of isolates tested, including a variety of different NTM species, and the use of a standardized inactivation and extraction procedure. However, our study failed to identify some clinically important species, such as the MAC and M. abscessus, directly from the positive MGIT broth. Some of the NTM species had limited numbers; this may lead to an insufficient database. Further studies are needed to verify the results in clinical practice.

In summary, our investigation revealed that the Vitek MS v3.0 system could be beneficial for clinical laboratories to identify commonly encountered NTM. We will focus on sample preparation to improve the identification rate in the future.

## MATERIALS AND METHODS

### Sample collection.

A total of 855 sequential NTM samples were collected from the Shanghai Pulmonary Hospital between March 2017 and March 2018. All the positive samples were considered clinically relevant, and the sources of the samples included sputum, bronchoalveolar lavage fluid, cerebrospinal fluid, urine, pleural fluid, and pericardial effusion. All samples submitted for mycobacterial cultures were inoculated into MGIT broth and cultured using the Bactec MGIT 960 instrument (Becton Dickinson, Cockeysville, MD, USA) and continuously monitored for 6 weeks. All positive culture isolates were confirmed by Ziehl-Neelsen acid-fast staining; they were also tested using an immunochromatographic assay with anti-MPT64 protein antibody (Hangzhou Genesis Biodetection & Biocontrol Ltd., China) to discriminate between the M. tuberculosis complex (MTBC) and NTM. All MPT64 assay-negative isolates were grown on Middlebrook 7H11 medium to evaluate the purity growth appearance and gene sequence used for identification. Identification of the clinical isolates used in this study was confirmed by DNA sequencing, which is considered to be the reference method for mycobacterial identification. For MALDI-TOF MS analysis, all sequencing-confirmed MGITs with positive signals were extracted for identification by following the sample preparation method.

### Sample preparation.

Culture broth (4 mL) was used for sample protocol methods to identify NTM. An Eppendorf tube was centrifuged at 13,000 × *g* for 10 min, and the supernatant was completely removed. Inactivation by mechanical disruption using 500-nm-diameter glass beads was performed. The bacterial pellet was resuspended in 500 μL of 70% ethanol and transferred to another 1.5-mL Eppendorf tube with 200 μL of 425- to 600-μm glass beads. The bacterial suspension was vortexed for 15 min and maintained at room temperature for 10 min for inactivating pathogens. Then the bacterial suspension was transferred to another 1.5-mL Eppendorf tube and centrifuged for 15 min to completely remove the supernatant. The dried pellet was mixed with 10 μL of 70% formic acid by vortexing for 3 to 5 s. Then, 10 μL of 100% acetonitrile was added and mixed by vortexing for 3 to 5 s. After centrifugation, 1.5 μL of the supernatant was transferred to the target slide. When the sample was dry, 1-cyano-4-hydroxycinnamic acid (CHCA) matrix was added and dried. The slide was loaded into the Vitek MS system for MALDI-TOF analysis.

### Quality control and calibration.

For instrument calibration, a reference strain of Escherichia coli (ATCC 8739) was transferred to the designated wells on the target slide, covered with a 1-μL Vitek MS-CHCA matrix, and air dried. The positive-control strain, M. smegmatis (ATCC 19420), was inactivated and extracted using the method described above. An analysis was performed at each test, together with the negative control (reagent alone).

### MALDI-TOF MS analysis.

The slide was run on a MALDI-TOF instrument (bioMérieux Vitek MS) to obtain the identification, and the results were analyzed using Knowledge Base (KB) v3.0. All organisms were placed in one well of a Vitek MS slide. Target plates were calibrated, and quality control was performed before and after data acquisition using E. coli (ATCC 8739). The Vitek result was considered accurate to the species level if a single identification was given and if it matched the identification obtained by the reference method. It was considered correct to the complex or group level if a single identification or multiple identifications within the same genus were reported and if these results were within the same complex or group as the species identified using the reference method. It was considered incorrect if a single identification was given that did not match the result obtained by sequencing at the species, complex, or group level. Identification was categorized as good (confidence level, ≥60.0% and <99.9%) or excellent (confidence level, ≥99.9%), and if the identification score was lower than 99%, a reanalysis was performed or the extraction was repeated. Repeat testing was performed with the original culture isolate, according to the procedures specified above. If an isolate still yielded no identification, this sample was considered an unidentified result. For results in which the confidence score was lower than 50%, repeated extraction analysis and sequencing were performed.

### Reference method of sequence-based typing.

The 16S rRNA and *hsp65* progressive retinal atrophy (PRA) genes were sequenced to identify isolates at the species level. DNA was extracted from 7H11 Middlebrook medium using a metal bath. A 20-μL mixture was prepared to amplify the PCR fragments. All analyzed strains were sequenced with the full 16S rRNA gene using the universal primers 27-FOR (AGAGTTTGATCMTGGCTCAG) and 1492-REV (TACGGYTACCTTGTTACGACTT). For species such as M. fortuitum, *M. peregrinum*, and *M. septicum*, *hsp65* PRA genes (FOR, ACCAACGATGGTGTGTCCAT, and REV, CTTGTCGAACCGCATACCCT) were used to supplement the identification. The result was considered correct if the isolate identified by Vitek MS KB v3.0 was consistent with the gene sequence. The sequencing result was considered the gold standard for discrepancies between sequencing and MALDI-TOF MS analysis.

### Reproducibility test.

Two operators performed the reproducibility test. A panel of three organisms (M. abscessus, M. chelonae, and M. smegmatis) was tested twice a day for 5 days. The operator was blinded to the identity of each organism. These tests were performed using three different reagents. The position of each organism on the target slide was determined in advance, and the organisms were randomly tested in sequence on one slide. The sample preparation, biological identification using Vitek MS v3.0, and analysis of the results were performed as described above.
